# Benthic and fish community composition on mesophotic reefs in Grand Cayman

**DOI:** 10.7717/peerj.17763

**Published:** 2024-08-29

**Authors:** Lucas Le Gall, Jack V. Johnson, Alex Chequer, Matthew Louis Doherty, Gretchen Goodbody-Gringley

**Affiliations:** Reef Ecology and Evolution Lab, Central Carribean Marine Institute, Little Cayman, Cayman Islands

**Keywords:** Mesophotic reef, Benthic, Carribean, Fish community, Cayman Islands, Mesophotic, Coral reef, Assemblages, Macroalgae, Benthic cover

## Abstract

Mesophotic Coral Ecosystems (MCEs) represent unique ecological habitats that range from 30 to 150 m deep, harbouring phylogenetically distinct species and offering refuge for many taxa during times of environmental stress. Yet owing to inaccessibility of ecosystems at these depths, most MCEs remain unexplored, with quantifications of ecological communities in these habitats lacking across many regions. Here, using open- and closed-circuit technical diving, we quantified benthic and fish community composition at four mesophotic reef sites (45 m depth) in Grand Cayman. We show significant differences in benthic community composition over a small spatial scale driven by disparate coverage of sponges, crustose coralline algae, and sand/rubble, yet consistent patterns of macroalgal dominance representing >50% coverage at each site and low hard coral cover at an average of 2.4%. Reef fish species richness, biomass, and density was consistent across sites, however the relative contribution of individual species to community composition differed significantly. Macrocarnivores were found to be the dominant contributors to biomass, with invertivores the most speciose, and omnivores and planktivores at the highest densities, consistent with previous descriptions of mesophotic fish assemblages in other regions. Similarly, the low hard coral cover and high macroalgae and sponge cover of the benthic communities also appear ecologically similar to several described mesophotic reefs yet is not uniform across the Caribbean. The ecological organisation of Grand Cayman’s MCEs may result from a variety of factors such as isolation from other major land masses, geology, local geography, and anthropogenic activity at both the local and global scale and highlight the importance of continued exploration and documentation of MCE communities.

## Introduction

Mesophotic Coral Ecosystems (MCEs) represent globally distributed, deeper water ecosystems, typically found between depths of 30 and 150 m (Pyle & Copus, 2019). Comprised of light-dependent scleractinian corals, these ecosystems also contain a variety of taxa including sponges, macroalgae, encrusting algae, and soft coral colonies (Hinderstein et al., 2010). Despite the ecological importance of MCEs for harbouring marine biodiversity, which contributes to coastal ecosystem services (Holstein et al., 2019), MCEs have historically received less attention than their shallow water counterparts due primarily to limited access using traditional SCUBA. However, as diving and submersible technology has advanced, so has access to the mesophotic, resulting in an increase in scientific investigation and biodiversity assessments (Pyle & Copus, 2019). This recent surge has primarily focused on Caribbean MCEs, which are typically located in regions with high anthropogenic activity, comprising roughly 55% of MCE focused publications as of 2018 (Laverick et al., 2018). These studies have been crucial in documenting the biodiversity of MCEs, highlighting the ecologically and biologically distinct nature of these systems (Kahng et al., 2010) with prominent differences in both benthic (Lesser, Slattery & Leichter, 2009; Stefanoudis et al., 2019) and fish assemblages (García-Sais et al., 2008; Lesser, Slattery & Leichter, 2009; Pinheiro et al., 2016; Stefanoudis et al., 2019) compared to shallow water ecosystems. Within MCEs, observed breaks in biodiversity are documented at approximately 60 m, distinguishing the upper and lower mesophotic zones (Lesser, Slattery & Leichter, 2009). While the defined depth range is influenced by topography, light intensity is also a major driving factor affecting community composition and defining species boundaries (Laverick, Andradi-Brown & Rogers, 2017; Pérez-Castro et al., 2022). Such physical factors of the environment differ among and within locations, however, resulting in strong differences in communities and distributions among various MCEs, warranting continued assessments of community composition.

The benthic composition of MCEs of the Cayman Islands has been previously described by Slattery & Lesser (2019), who focused primarily on sponge communities over limited spatial distribution and document a “sponge belt” (Slattery & Lesser, 2019). Mesophotic sponges in Grand Cayman were also the focus of Macartney et al. (2021), whose work was concentrated on a single species. While Carpenter et al. (2022) assessed the benthic composition of corals and key functional groups on MCEs across Little Cayman Island, their study did not include Grand Cayman. Importantly, none of the previous mesophotic studies in the Cayman Islands included assessments of fish communities, nor the interaction of benthic habitat and fish assemblages, highlighting a critical knowledge gap in our understanding of these ecosystems in Grand Cayman.

Variations in benthic habitat often corresponds to changes in fish community composition. For example, in the Seychelles shallow reefs with high macroalgal cover had higher densities of generalist fish species and lacked specialists, whereas reefs with high coral cover had higher abundances of specialised corallivores species (Chong-Seng et al., 2012). In the Philippines, a positive association was found between hard coral cover and biomass of planktivores, piscivores, and corallivores, while a negative association was found between hard coral cover and detritivores and sand feeders (Russ et al., 2021). In the Caribbean, a study found a positive correlation between macroalgae density and herbivorous fish assemblages on shallow reefs but fail to show an impact of coral cover on fish populations (Sandin, Sampayo & Vermeij, 2008). Mesophotic studies in the Gulf of Mexico have also shown a difference of fish assemblages in relation with benthic composition, with high fish biomass associated with coralline algal reefs (Voss et al., 2014). As these patterns vary across locations and depths, it is important to include assessments of both benthic habitat and fish communities to accurately characterize MCEs, which was previously lacking for MCEs in Grand Cayman.

Here, we characterize the biodiversity and community composition of mesophotic reefs across four sites on Grand Cayman Island. Using benthic photogrammetry and *in situ* visual surveys we provide a description of the benthic community composition, including scleractinian corals and key functional groups, as well as of fish community assemblages. Given the hypothesized role of MCEs as areas of refuge for preserving biodiversity under future climate change scenarios (*e.g*., DRRH; Bongaerts et al., 2010), baseline characterizations of these habitats are crucial for increasing our understanding of mesophotic ecosystem function and informing marine spatial planning and management.

## Materials and Methods

**Figure 1:**
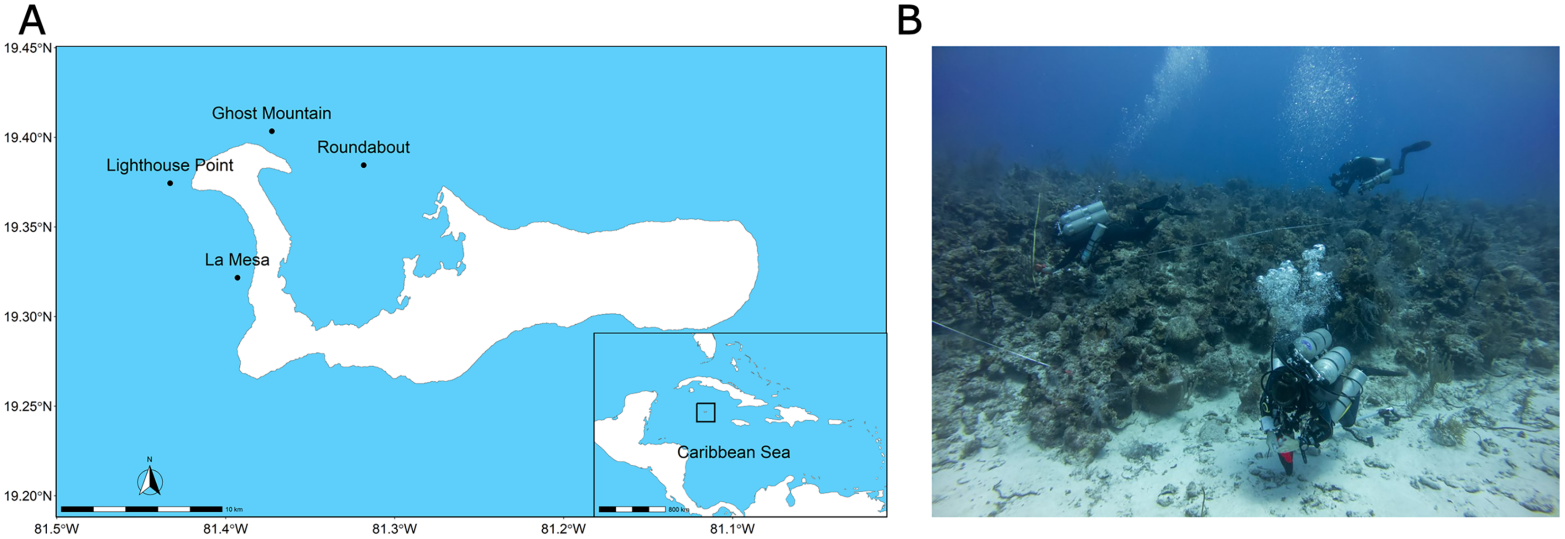
Context of the study and sampling techniques. Maps of the study sites, Lighthouse Point, Ghost Mountain, La Mesa and Roundabout and the position of Grand Cayman amidst the Caribbean Sea (A). Picture of the dive team laying transect and taking pictures of the quadrat during a 15 m practice dive (B). Site map generated with the “rnaturalearthdata” package (https://cran.r-project.org/package=rnaturalearth). Photo credit: Alex Chequer.

The present study was carried out off the coast of Grand Cayman (19.329858–81.252361), the biggest and most populated of the three islands that compose the Cayman Islands (Economics and Statistics Office Government of the Cayman Islands, 2022). Four sites situated around the West Bay peninsula were surveyed: Ghost Mountain (19.402300–81.385680), Roundabout (19.384333–81.318300), La Mesa (19.321680–81.393080), and Lighthouse Point (19.372954–81.421855) (Fig. 1). The sites were situated off the wall, with a steep slope except for lighthouse point that is characterized by a more gradual slope (G. Goodbody-Gringley, 2022, personal observation). All four sites were composed of alternating hard-bottom and sandy substrates.

Fish surveys were conducted along 30 m by 2 m belt transects at a depth of 45 m (*n* = 3–5 per site; 60 m^2^ per transect). All fishes encountered along the transects were identified to species level, counted, and categorised into size classes based on total length: 0–5, 5–10, 10–20, 20–30, 30–40, and >40 cm. Fish surveys were conducted exclusively by divers using closed-circuit rebreathers (Hollis Prism2) to limit any influence of open *vs*. closed circuit diving on fish behavior. Following the surveys, fish data were classified into one of five food web classes: herbivore, invertivore, omnivore, macrocarnivore, and planktivore based on data available on www.fishbase.org (Welcomme, 1988; Houde & Zastrow, 1993), a data base by Froese & Pauly (2000). Density was calculated as the total number of individuals per transect (no. of fish/60 m^2^ transect). Biomass was calculated using the formula:

$${{\left( {a \times {Lb}} \right)} \over N}$$

where L is the estimated length using the upper end of the size class category, N is the number of individuals of that size class (Bohnsack & Harper, 1988; Kulbicki et al., 1993), parameter $a$ is a scaling constant varying between species and parameter b is an allometric scaling factor. When b = 1, it indicates isometry and biomass scales linearly with size, when b < 1, it indicates negative allometry, and biomass increase slower than size and when b > 1, it is positive allometry and biomass increases faster than size. Values for a and b were obtained for each species on www.fishbase.org (Welcomme, 1988, Houde & Zastrow, 1993). When these values were not available for a particular species, data for a congeneric species were used. Lastly, species richness is the total number of species per transect and diversity is based on the Shannon index of diversity (Shannon & Weaver, 1949):

$$- \sum{{\rm P}_i}\left( {{\rm log}{{\rm P}_i}} \right)$$

with P_i_ being the proportion of each species in the sample.

At each of the four sites, a 10 m × 10 m plot was laid on the benthos using transect tapes, with the lower edge at 45 m depths. Images of the benthos were taken every second using the time lapse setting by two divers that swam in a lawnmower pattern approximately 1 m above the benthos, using two GoPro Hero 10 cameras mounted onto a PVC frame with two lights (Sola Video 2500 Flood). Eight targets were laid haphazardly on the substrate within the plot to assist with building a digital 3D model of the studied area using the software AGIsoft Metashape Pro (version 1.8.3). This was then converted into a 2D orthomosaic and exported as a PNG that was subsequently split into ~1 m^2^ replicate quadrats (48 in Ghost Mountain, 90 in La Mesa, 99 in Lighthouse Point and 100 in Roundabout) using Photoshop (version 24.3) and scaled on the pictured measuring tape. ImageJ (version 1.53t) software was then used for further analysis on all 337 images. Within each quadrat all scleractinian corals were identified to the highest taxonomic level possible based on image quality. Species within the genera *Agaricia*, *Siderastrea*, and *Orbicella*, were limited to genus. Surface area of each colony was then measured using the freehand tool (in cm^2^). Total surface area of all corals relative to the size of the quadrat was then used to calculate percent coral cover.

Benthic composition of other key functional groups was then assessed visually to the nearest 1% cover for eight categories: Crustose Coralline Algae (CCA), Soft Coral (SC), Sand and Rubble (S/R), Turf Algae (TA), Recently Dead Coral (RDC), Macroalgae (MA), and Other (soft coral plumes, fish, targets, blurry and indistinguishable area, *etc*.). Measurements and visual assessment of the functional groups were conducted by the same person throughout the data collection phase to limit bias and errors. Statistical analyses were conducted on R version 4.1.0 (R Core Team, 2023).

Fish density, biomass, diversity, coral diversity index and richness were compared among sites and among food webs with non-parametric Kruskal-Wallis’ tests (Kruskal & Wallis, 1952), as they did not meet the assumption of normality (Shapiro & Wilk, 1965), followed by *post-hoc* analysis using the Dunn’s test (Dunn, 1964) in the FSA package (Ogle et al., 2023). The fish diversity index met the assumption of normality (Shapiro & Wilk, 1965) and was compared among sites with a one-way analysis of variance (ANOVA), followed by a *post hoc* analysis using Tukey’s test (Tukey, 1949).

Benthic functional group composition among sites did not meet the assumptions of normality (Shapiro & Wilk, 1965) and was therefore compared using a Kruskal Wallis with a Dunn’s test as a *post hoc*. “Other”, while being measured as a functional group, is not integrated into any of the mentioned statistical analyses. By being an imprecise category that is compensating for a various array of non-benthic category measures or photomosaic inconsistence, it was judged irrelevant to the aim of the study and the measured percentages have been scaled accordingly.

To assess dissimilarity in the benthic composition and fish community composition among sites, we used non-Metric Multi-Dimensional scaling (nMDS) models from the Vegan package (Oksanen et al., 2022). Models were based on a Bray-Curtis dissimilarity matrix of community composition for both benthic composition, fish species composition, and reef fish trophic community. A Bray-Curtis dissimilarity is less sensitive to the absence of species within the matrix compared to the distance (metric) based counterparts. Thus, a dissimilarity matrix is preferable for our dataset which reflects community composition rather than a distance matrix that explains the distance between points without assessing the difference in community composition (Jaccard, 1901). Subsequently, to statistically compare the dissimilarity between sites for community composition, we used a PERMANOVA test where we set the dissimilarity matrix as the response, and site as the predicting factor.

## Results

**Figure 2:**
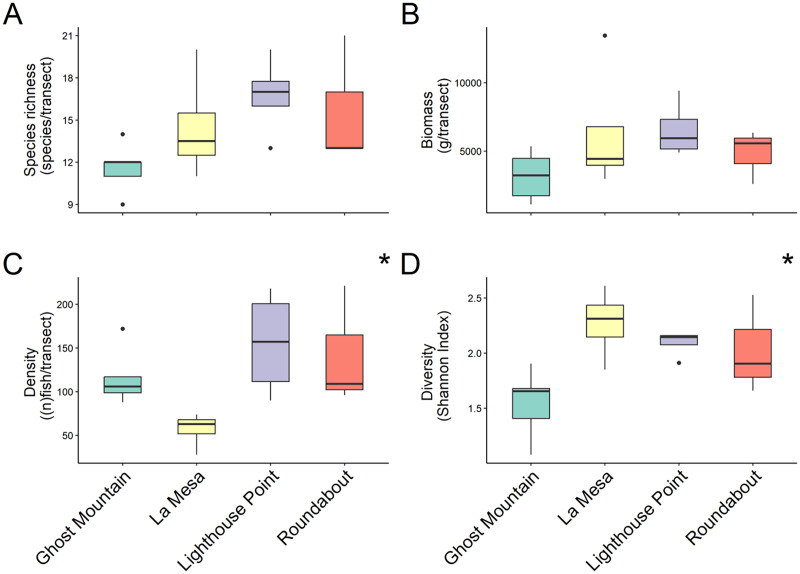
Comparison of species richness (number of unique species per transect) (A), biomass (estimated weight of fish in g per transect) (B), density (number of fish per transect) (C) and diversity (Shannon diversity index) (D) between sites. Boxplots’ horizontal bars represent the sites median values, the upper and lower sections (box outlines) represent the interquartile range, the whiskers extend to 90% of the data, with dots being outliers. Biomass is calculated using the formula (a * L^b^)/N where a is a scaling constant different for each species, b is an allometric scaling factor that is different for each species, L is the length of the individual in cm and N is the number of individuals of the species. The diversity value is obtained *via* the Shannon-Weaver index of diversity (Shannon & Weaver, 1949). Based on Kruskal Wallis’ test followed by a Dunn’s test as *post hoc*, median species richness and biomass among sites did not differ significantly (Kruskal & Wallis, 1952). Median fish density between Lighthouse Point and La Mesa was significantly different (X^2^ = −2.74, *P* = 0.03), as well as the Shannon-Weaver diversity index between Ghost Mountain and La Mesa (X^2^ = 0.726, *P* = 0.019). *Significant difference (*P* < 0.05).

In total 48 fish species were recorded across all four sites ranging from 30 at Lighthouse Point to 24 at Roundabout, however, median species richness per transects did not differ significantly among sites (chi-squared = 5.803, *P*-value = 0.1216; Kruskal Wallis) (Fig. 2A). Yet, according to the species accumulation curve, the number of surveys conducted was not enough to account for the full species composition, and thus this is likely an underestimate of diversity (Fig. S1). Fish biomass also did not differ significantly among sites (chi-squared = 4.6559, *P* = 0.1988; Kruskal Wallis) (Fig. 2B), Lighthouse Point had the highest median biomass (5,936.296 ± 1,026.23 g/transect), while Ghost Mountain had the lowest (3,211.59 ± 797.70 g/transect) (Fig. 2B). The median fish density was significantly different across sites (chi-squared = 9.1522, df = 3, *P*-value = 0.02733; Kruskal Wallis). Among sites, the median fish density only presented significant differences (X^2^ = −2.74, *P* = 0.03; Dunn’s Test) between Lighthouse Point (157 ± 30.39 fish per transect) and La Mesa (63 ± 10.08 fish per transect) (Fig. 2C). Shannon diversity index was significantly different among sites (F-value = 4.655, *P*-value = 0.022; ANOVA) between Ghost Mountain and La Mesa (X^2^= 0.726, *P* = 0.019; Tukey), being respectively the least diverse with a median index value of 1.65 ± 0.14 and the most diverse with a median value of 2.31 ± 0.15 (Fig. 2D).

**Figure 3:**
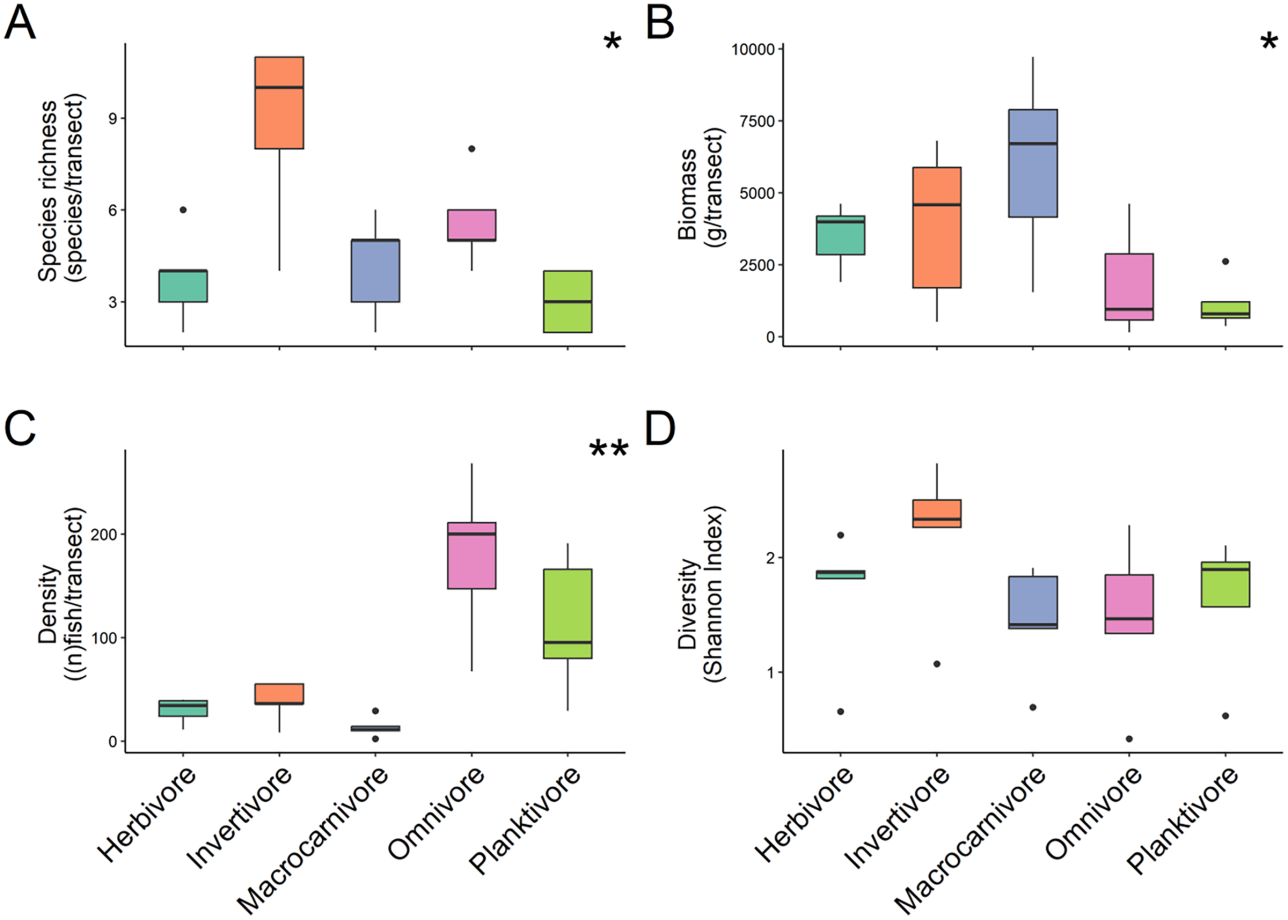
Comparison of species richness (number of unique species per transect) (A), biomass (estimated weight of fish in mg per transect) (B), density (number of fish per transect) (C), and diversity (Shannon diversity index) (D) between trophic guilds. Boxplots’ horizontal bars represent the sites median values, the upper and lower sections (box outlines) represent the interquartile range, the whiskers extend to 90% of the data, with dots being outliers. Biomass is calculated using the formula (a * L^b^)/N where a is a scaling constant different for each species, b is an allometric scaling factor that is different for each species, L is the length of the individual in cm and N is the number of individuals of the species. The diversity value is obtained *via* the Shannon-Weaver index of diversity (Shannon & Weaver, 1949). Based on Kruskal Wallis’ test followed by a Dunn's test as *post hoc*, species richness (X^2^ = 12.169, *P* = 0.016), fish density (X^2^ = 17.031, *P* = 0.001) and biomass (X^2^ = 9.622, *P* = 0.047) among trophic guilds were significantly different. However, the Shannon diversity index did not differ significantly when comparing trophic groups. Significant difference *P < 0.05, **P < 0.005.

For trophic guilds specifically, invertivores were the most species rich group of fish with a median of 10 ± 1.31 species recorded and planktivores were the least with a median species richness of 3 ± 0.4 (Fig. 3A). Macrocarnivores had the highest median biomass (6,693.41 ± 1,434.09 g/transect; Fig. 3B) while planktivores had the lowest median biomass (789.6238 ± 394.81 g/transect). Omnivores had the highest median density (200 ± 4.42 of fish per transect; Fig. 3C). Significant differences were found in species richness (X^2^ = 12.169, *P* = 0.016; Kruskal Wallis) (Fig. 3A), biomass (X^2^ = 9.622, *P* = 0.047; Kruskal Wallis) (Fig. 3B) and fish density (X^2^ = 17.031, *P* = 0.001; Kruskal Wallis) (Fig. 3C) among trophic guilds. Higher species richness was observed for invertivores compared to planktivores (X^2^ = 3.13, *P* = 0.017; Dunn’s Test), while macrocarnivores had significantly lower density compared to planktivores (X^2^ = −2.816, *P* = 0.043; Dunn’s Test) and omnivores (X^2^ = −3.697, *P* = 0.002; Dunn’s Test). Shannon diversity index values did not differ significantly (F-value = 1.236, *P* = 0.327, ANOVA) (Fig. 3D) among trophic groups.

**Figure 4:**
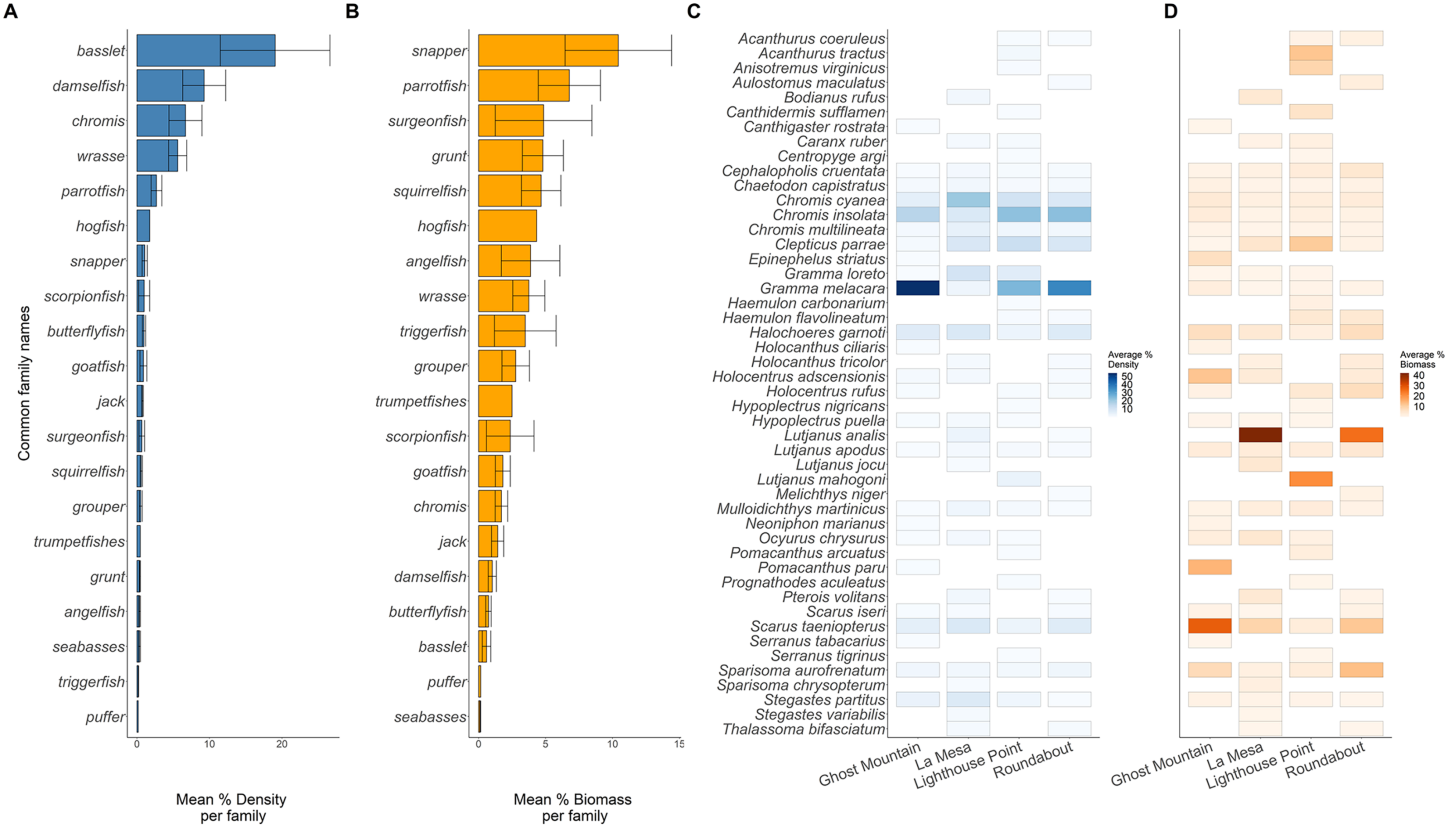
Biomass (A) and density (B) of the common fish families across all sites. Biomass (C) and density (D) of each species across all sites. Solid lines in plots (A and B) are the average per common family, and the whiskers are the range of the data. Each rectangle in (C and D) shows at least one occurrence of a species on one of the transects. The absence of a rectangle represents the absence of a species on a site.

Across sites, 20 families of fish were recorded (Fig. 4). The basslet family (*Grammatidae*), had the highest density per square meter with a mean value of 19.048 ± 7.554 % (Fig. 4A), but only accounted for 0.59 ± 0.317 % of the total fish biomass (Fig. 4B). Members of the snapper family (*Lutjanidae*) had the highest average biomass (10.432 ± 3.974 %) but only accounted for 0.071 ± 0.031% of the recorded density (Figs. 4A and 4B).

Across the surveyed sites, *Gramma melacara*, had the highest frequency by density at three sites, with 53.78% at Ghost Mountain, 35.91% at Roundabout and 24.75% at Lighthouse Point, while *Chromis cyanea* had the highest frequency at La Mesa of 20.61% (Fig. 4C). *Scarus taeniopterus* had the highest frequency by biomass at Ghost Mountain at 28.02%, while *Lutjanus analis* had the highest frequency by biomass at La Mesa at 41.85%, and Roundabout at 25.46% (Fig. 4D). At Lighthouse Point, *Lutjanus mahogoni* had the highest frequency by biomass at 20.91% (Fig. 4D). Out of the 48 observed species, only 13 were found across all sites and 22 species were endemic to one site.

**Figure 5:**
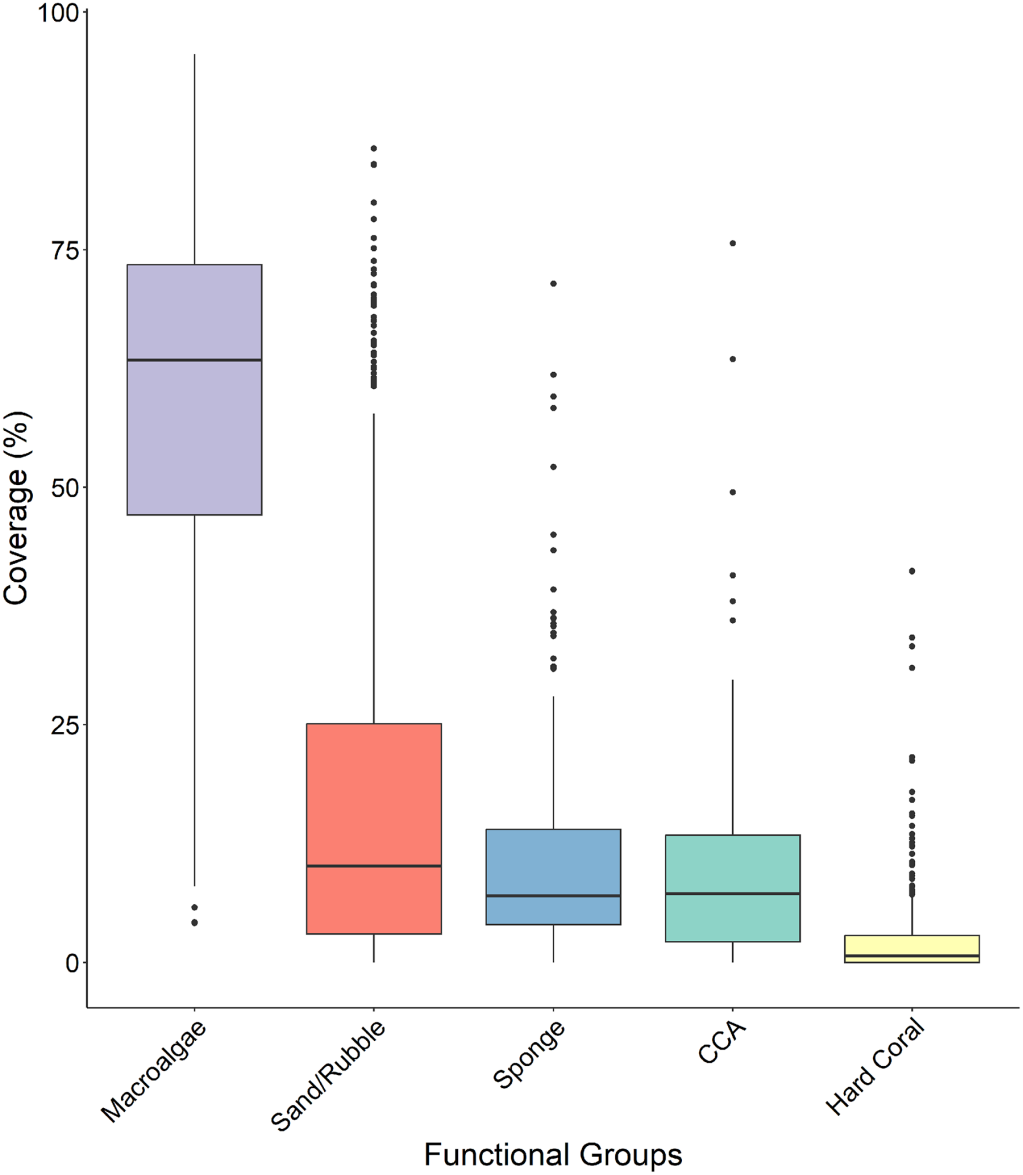
Comparison of the percentage cover of the different functional groups (macroalgae, sand/rubbles, sponge, CCA and hard coral). The horizontal middle bars represent the median values, whilst the upper and lower sections (box outlines) represent the interquartile range. The whiskers extend to 90% of the data, with dots being outliers. Each functional group was compared using a Kruskal Wallis test followed by a Dunn’s test, as the values did not meet the assumptions of normality.

Macroalgae percent cover was significantly higher than all other functional groups across all four sites (Fig. 5 and Table 1) with a median surface coverage of 63.378 ± 1.125%, while hard coral cover was significantly lower than all other functional groups, with a median of 0.662 ± 0.285% (Fig. 5 and Table 1). Sponge cover did not differ significantly from either sand/rubble or CCA cover, however, sand/rubble cover was significantly higher than CCA (Fig. 5 and Table 1).

**Table 1:** Benthic component’s adjusted *P*-values throughout sites.

	Sand/rubble	Sponge	CCA	Hard coral
Macroalgae	<0.001	<0.001	<0.001	<0.001
Sand/rubble		0.067	0.001	<0.001
Sponge			0.157	<0.001
CCA				<0.001

**Figure 6:**
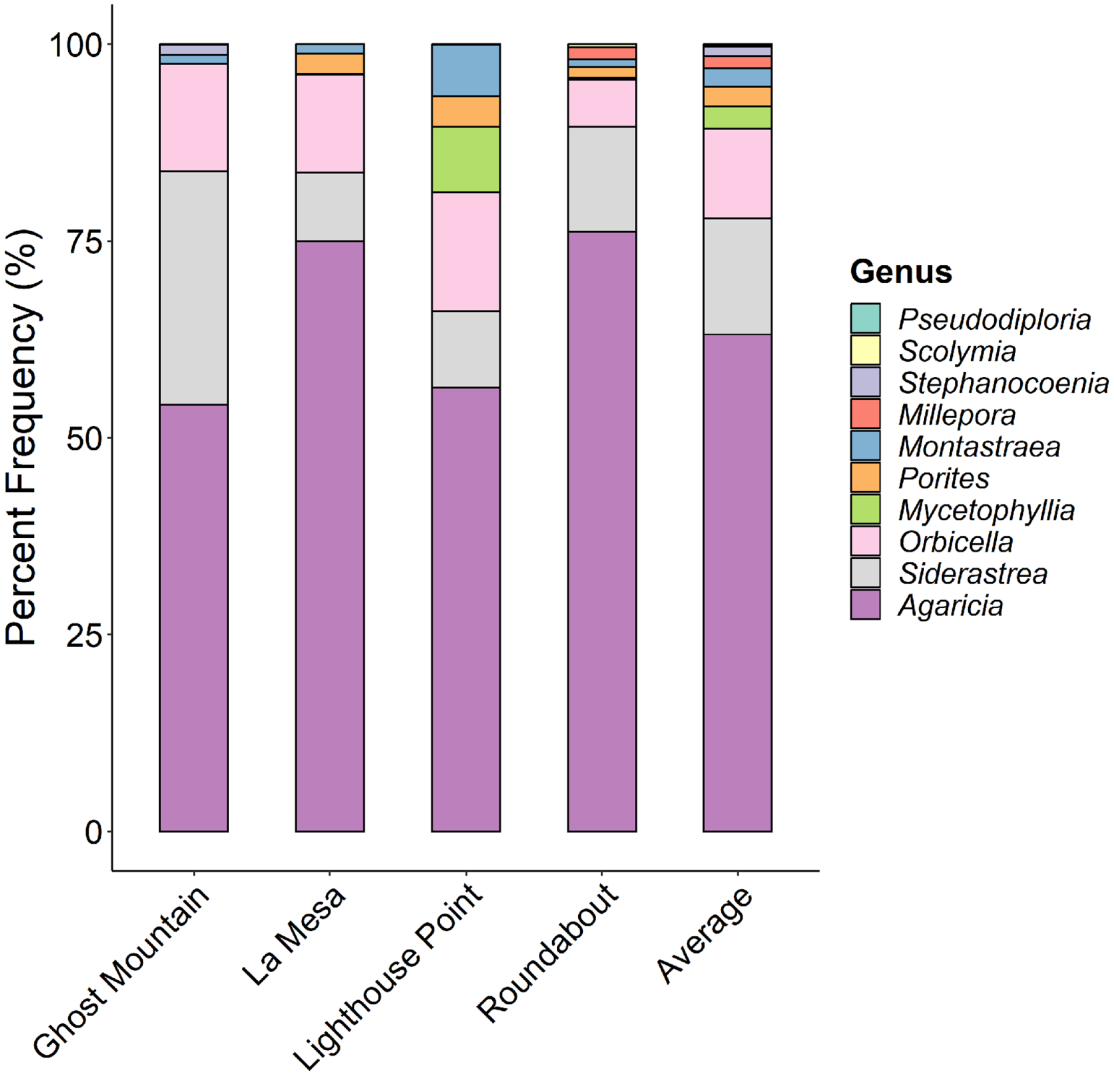
Comparison of the percent contribution of each Scleracctinian coral genus observed per site. With Agaricia being the most common genus recorded across all sites representing up to 70% of the coral cover at Ghost Mountain.

*Agaricia* was the dominant coral genus at all four sites (Fig. 6) ranging from 77% at Ghost Mountain to 54% at Lighthouse Point. *Siderastrea* and *Orbicella* were present in moderate frequency at all sites, *Montastraea* and *Porites* were consistently present but in low coverage, and *Mycetophyllia* was only recorded in La Mesa. Shannon diversity of coral species showed no significant differences among sites (*P* > 0.05, Kruskal-Wallis).

**Figure 7:**
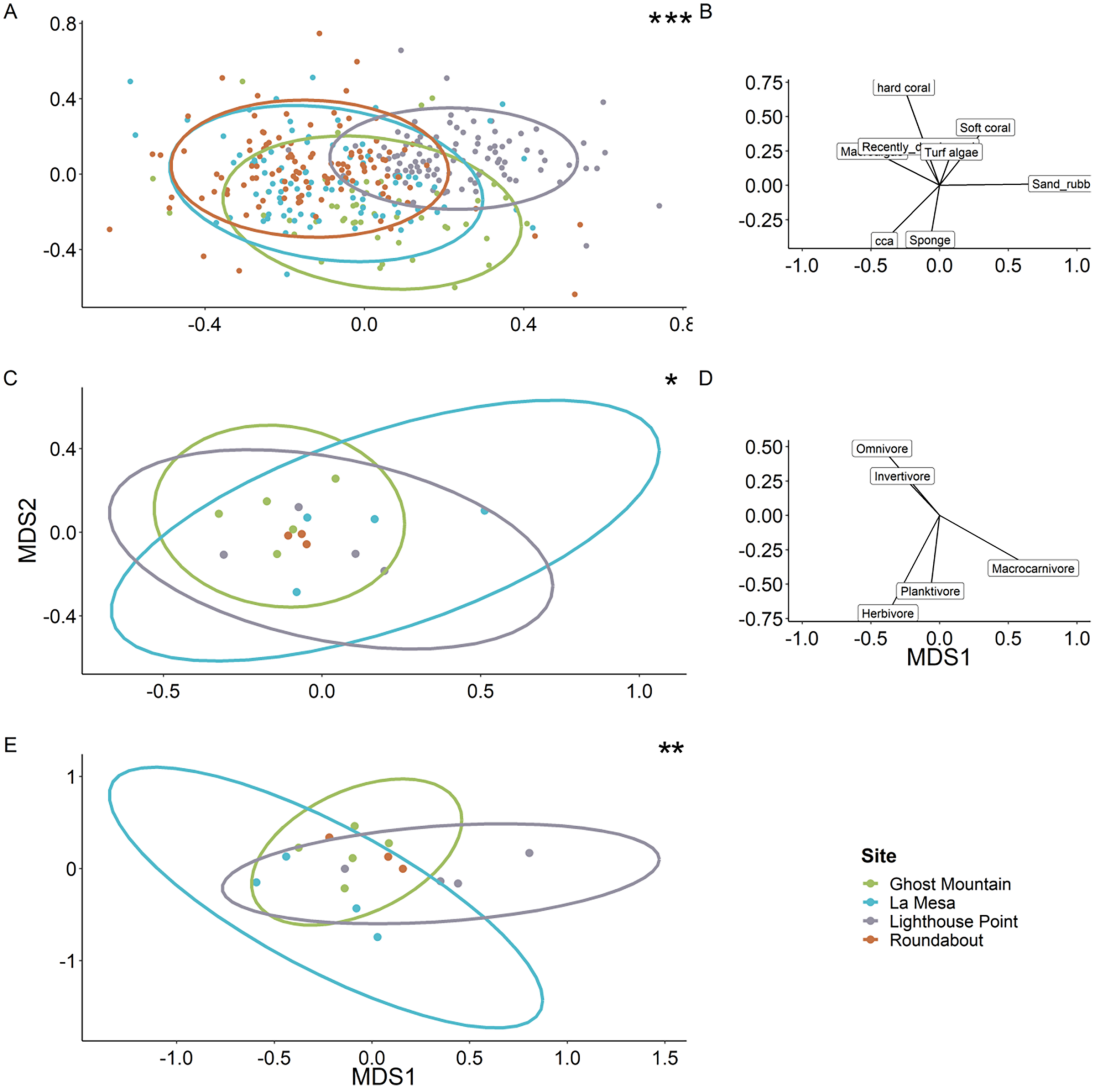
Ordination plots of the dissimilarity between the sites regarding benthic composition (A), fish trophic guild composition (C) and fish species (E). Each dot represents a quadrat for the benthic ordination plot (A) and a transect for the fish trophic plots (C and E). Roundabout’s ellipse is not present owing to a deficit of replicates which does not allow the projection (C and E). Each ellipse represents the tendency of a site to drift along the two axes following the vectors on the right of each ordination plot (B and D). When compared *via* a multivariate analysis, it reveals a significant difference of benthic composition among sites. It also showed a significant difference between fish communities (PERMANOVA, F value = 2.76, *P* = 0.0058) and species composition among sites (PERMANOVA, F value = 2.55, *P* = 0.003). Significant difference *P < 0.05, **P < 0.005, ***P < 0.0005.

Multivariate analyses revealed the benthic composition was significantly dissimilar among sites (PERMANOVA, F value = 11.69, *P* = 0.0001). Lighthouse Point appears to be the most unique site based on benthic composition (Fig. 7A), related to high sand/rubble coverage (Fig. 7B). The trophic composition of fish communities (Fig. 7C) was also significantly different among sites (PERMANOVA, F value = 2.76, *P* = 0.0058) as was the species composition (Fig. 7E; PERMANOVA, F value = 2.55, *P* = 0.003).

## Discussion

Here, we provide the first characterization of mesophotic reefs in the Cayman Islands that combines benthic habitat and fish assemblages. Our results show that fish populations are primarily composed of basslet (*Grammidae*) and damselfish (*Pomacentridae* > *Stegastinae*) species, with species of wrasse (*Labridae*) and chromis (*Pomacentridae* > *Chrominae*) also present in high relative abundance. Although present in lower relative densities, species of snapper (*Lutjanidae*) and parrotfish (*Scaridae*) represent the highest contribution to community biomass on the mesophotic reefs surveyed (Fig. 4). The benthos at 45 m is dominated by macroalgae and sand/rubble, which can be generally described as algae-covered hard substrate dispersed among patches of sand and rubble. Sponges, seafans, and crustose coralline algae are also found in high frequency. However, hard coral cover represents the lowest percent cover (Fig. 5). Among the corals present, the dominant contributing species belong to the genera *Agaricia, Siderastrea*, and *Orbicella* (Fig. 6).

Upper mesophotic reefs often represent transition zones between shallow water ecosystems and lower mesophotic ecosystems (Pyle & Copus, 2019). Fish populations in these regions frequently harbor both depth-generalist species as well as distinctive mesophotic fauna (Baldwin, Tornabene & Robertson, 2018; Bejarano, Appeldoorn & Nemeth, 2014; Pinheiro et al., 2016). In fact, many of the species found on the MCEs surveyed here overlap with those documented on neighboring shallow reef sites in Grand Cayman (Johnson, Chequer & Goodbody-Gringley, 2023). Yet, among all the mesophotic sites surveyed, *Gramma melacara* (blackcap basslet) and *Chromis insolata* (sunshinefish) are the most abundant in terms of number of fish, species that are absent on nearby shallow reefs (Johnson, Chequer & Goodbody-Gringley, 2023). Previous surveys of Caribbean fish communities also note high densities of *C. insolata* on mesophotic reefs with their presence generally lacking on shallow reefs, suggesting this species is a mesophotic specialist (Pinheiro et al., 2016; Baldwin, Tornabene & Robertson, 2018; García-Hernández et al., 2018; Gress et al., 2018). Likewise, *G. melacara* is also a mesophotic specialist and is considered common on MCEs in the Western/Central Caribbean (Dustan & Lang, 2019; Chasqui et al., 2020), however it is not present on MCE species lists from Cozumel (Gress et al., 2018), Curacao (Pinheiro et al., 2016; Baldwin, Tornabene & Robertson, 2018), or Bermuda (Pinheiro et al., 2016; Goodbody-Gringley et al., 2019), and thus its geographic distribution may be limited to the Central Caribbean. Interestingly, despite being abundant in other locations at this depth (Andradi-Brown et al., 2017; Goodbody-Gringley et al., 2019, 2023) very few invasive lionfish (*Pterois volitans*) were found in this study (two total), suggesting they are currently not a major component of the mesophotic fish populations in Grand Cayman. Overall, the upper mesophotic reefs of Grand Cayman can clearly be categorized as transition ecosystems as they house both depth-generalist and mesophotic specialist species, however, species richness, diversity and abundance were all lower on MCEs (this study) compared to shallow reefs (Johnson, Chequer & Goodbody-Gringley, 2023).

Although species of snapper (*Lutjanidae*) are the most abundant by biomass across all sites, this is not consistent among the four sites surveyed, with these species being generally absent from Ghost Mountain. Rather, parrotfish are the dominant group at this site, where the most abundant species by biomass is *Scarus taenopterus*. Within the other three sites, variation also exists among the dominant species of snapper with *Lutjanus analis* being the highest contributor to biomass at La Mesa and Roundabout, while *Lutjanus mahogoni*, is the highest contributor at Lighthouse Point. Macrocarnivores, such as snappers, are often transient. So, it is likely that the variations in species contributions of these groups and the absence of snappers from surveys at Ghost Mountain (Fig. 4) may be an artifact of the visual survey method, which is limited to a short period of time and may miss transient species. Fish density and richness was found to differ significantly among sites, being higher in Lighthouse Point compared to the other sites. Lighthouse Point is situated at the northwest corner of Grand Cayman and is often characterized by converging currents that lead to strong mixing and potential upwelling. Increased mixing promotes nutrient availability and fuels the food chain, potentially leading to the significantly higher density and more speciose fish population found at this site. Increased plankton availability due to mixing is supported by the significantly higher density of planktivores compared to other trophic guilds found at Lighthouse Point (Bodungen et al., 1982; Rosa et al., 2015). Biomass, however, does not differ among sites, indicating that although density is higher at Lighthouse Point the average fish size is smaller. Smaller fish at this site may be related to the unique benthic composition of Lighthouse Point, which was dominated by areas of sand/rubble with low rugosity, limiting habitat availability for larger-bodied fishes (Kuffner et al., 2007).

Benthic cover of MCEs in Grand Cayman was found to be similar to mesophotic reefs at other Central Caribbean locations (Carpenter et al., 2022; Dustan & Lang, 2019; Reed et al., 2019). For example, sponges are the highest contributing benthic invertebrate to percent cover on MCEs in Grand Cayman, constituting an average of 10.587 ± 0.560% of the benthos, which conforms to previous reports from Lesser, Slattery & Mobley (2018) who described an increase in sponge abundance and diversity with increasing depth. However, while macroalgae cover in Grand Cayman is similar to that on MCEs at Pulley Ridge (Florida, USA) and in Puerto Rico (Reed et al., 2019; Appeldoorn et al., 2019), it is higher than that reported for several other Caribbean MCEs (Dustan & Lang, 2019; Gress et al., 2019), including Little Cayman (Carpenter et al., 2022). Differences in macroalgae cover between these neighbouring islands may be related to differences in levels of marine protection, as roughly 57% of the nearshore environment in Little Cayman is classified as no-take marine protected areas compared to less than 20% in Grand Cayman. While comparable fish data for mesophotic reefs in Little Cayman is not available, protection of ecologically important reef fish populations, such as herbivores, is expected to lead to reduced algal cover (Jackson et al., 2014).

While coral cover at 45 m depth in Grand Cayman is low, at an average of 2.6%, similar percent coral cover is noted on MCEs in Honduras, Mexico, and at Pulley Ridge. Yet, it is lower than the 8% cover documented in Bonaire and substantially lower than in Puerto Rico and the US Virgin Islands, where percent cover reaches nearly 30% on upper mesophotic sites (Appeldoorn et al., 2019; Frade et al., 2019; Gress et al., 2019; Reed et al., 2019; Smith, Holstein & Ennis, 2019). Despite regional differences in percent cover of scleractinian corals, species presence on MCEs appears relatively homogenous across the Caribbean, with species in the genus *Agaricia* being predominant (Reed et al., 2019; Gress et al., 2019; Appeldoorn et al., 2019; Smith, Holstein & Ennis, 2019; Frade et al., 2019). In Grand Cayman, coral species assemblages closely resemble those of nearby Little Cayman, where species in the genera *Agaricia, Siderastraea* and *Orbicella* have the highest contribution to percent cover at 45 m depth (Carpenter et al., 2022). However, *Madracis spp*. occur in relatively high frequency in Little Cayman yet are absent from the surveys in Grand Cayman. Conversely, the genus *Mycetophylia* is a top contributor to MCEs in Grand Cayman but not in Little Cayman. Variations in percent coral cover and species contributions among locations are likely driven by the availability of incident light as a function of reef topography. For example, on the *Orbicella* reefs in the US Virgin Islands, the reef slope is gradual with extensive flat regions in the upper mesophotic zone (Smith, Holstein & Ennis, 2019). However, the Cayman Islands are surrounded by deep trenches, with near vertical walls beyond the shallow fore reef leading to limited light availability beyond 30 m (Lesser, Slattery & Mobley, 2018; Slattery & Lesser, 2019).

Alternatively, differences in benthic composition, such as the dominance of *Agaricia spp* on Grand Cayman MCEs, may be related to disturbance history. The proximity of these MCEs to the Island of Grand Cayman exposes these sites to heavy levels of tourism and overfishing, impacting both coral and fish communities (Stallings, 2009; Johnson, Chequer & Goodbody-Gringley, 2023). In fact, benthic composition of MCEs is documented to affect the fish community, with high coral cover being linked to high fish density and richness (Garcia-Sais, 2010). Thus, MCEs likely do not provide a refuge from disturbance for coral and fish communities in regions with high disturbance frequency or severity, and will likely be composed of weedy, fast-growing coral species that can tolerate high disturbance conditions, associated with less dense and less diverse fish populations.

## Conclusion

In conclusion, this study provides an overview of benthic and fish assemblages of Grand Cayman’s mesophotic reefs at 45 m, which qualitatively appear different to other regions within the Caribbean (Smith et al., 2010; García-Hernández et al., 2018; Scott et al., 2019). Our study shows that benthic composition is highly variable across a small geographical scale, with the exception of macroalgae, being the dominant benthic component across all sites. Fish assemblages were also distinct, with representatives of both depth-generalist and mesophotic-specific species present at all sites, but differences in biomass and density of trophic guilds among sites. Whether the unique composition of benthic and fish communities is a result of geological and biological processes, or the influence of local scale anthropogenic activity remains to be seen. Future research on remote mesophotic reefs within the same ecoregion will provide further insight into why Grand Caymans mesophotic reefs appear as distinct communities and will allow assessments of the relative influence from local anthropogenic activity.

## Supplemental Information

10.7717/peerj.17763/supp-1 Supplemental Information 1 Species, Biomass and number of fish. The dataset and code used to create Figures 2, 3, 4 and a part of Figure 7 as well as for the data analysis.

10.7717/peerj.17763/supp-2 Supplemental Information 2 Functional group percent cover throughout sites. The dataset and the codes used to create Figures 5 and 7 and Table 1 and for the data analysis.

10.7717/peerj.17763/supp-3 Supplemental Information 3 Species accumulation curve comparing the cumulative count of observed species over the total number of surveys. The curve did not reach a plateau after 16 surveys, showing the need for more replicates of the study. Light purple zone is the confidence interval around the mean with a level of confidence of 95%.

10.7717/peerj.17763/supp-4 Supplemental Information 4 Map of Grand Cayman and the Carribean. Code used to create Figure 1.

10.7717/peerj.17763/supp-5 Supplemental Information 5 Size and genus of all the recorded coral colonies throughout sites. The dataset and the codes used to create Figure 6 and for the data analysis.

## Additional Information and Declarations

### Field Study Permissions

The following information was supplied relating to field study approvals (*i.e*., approving body and any reference numbers): We did not need permits for this work.

### Data Availability

The following information was supplied regarding data availability: The raw data is available in the Supplemental Files.
