# Supplementary figures and images for: Benthic and fish community composition on mesophotic reefs in Grand Cayman
Source: PeerJ. 2024 Aug 29;12:e17763. doi: 10.7717/peerj.17763 (PMC11366224; doi:10.7717/peerj.17763)

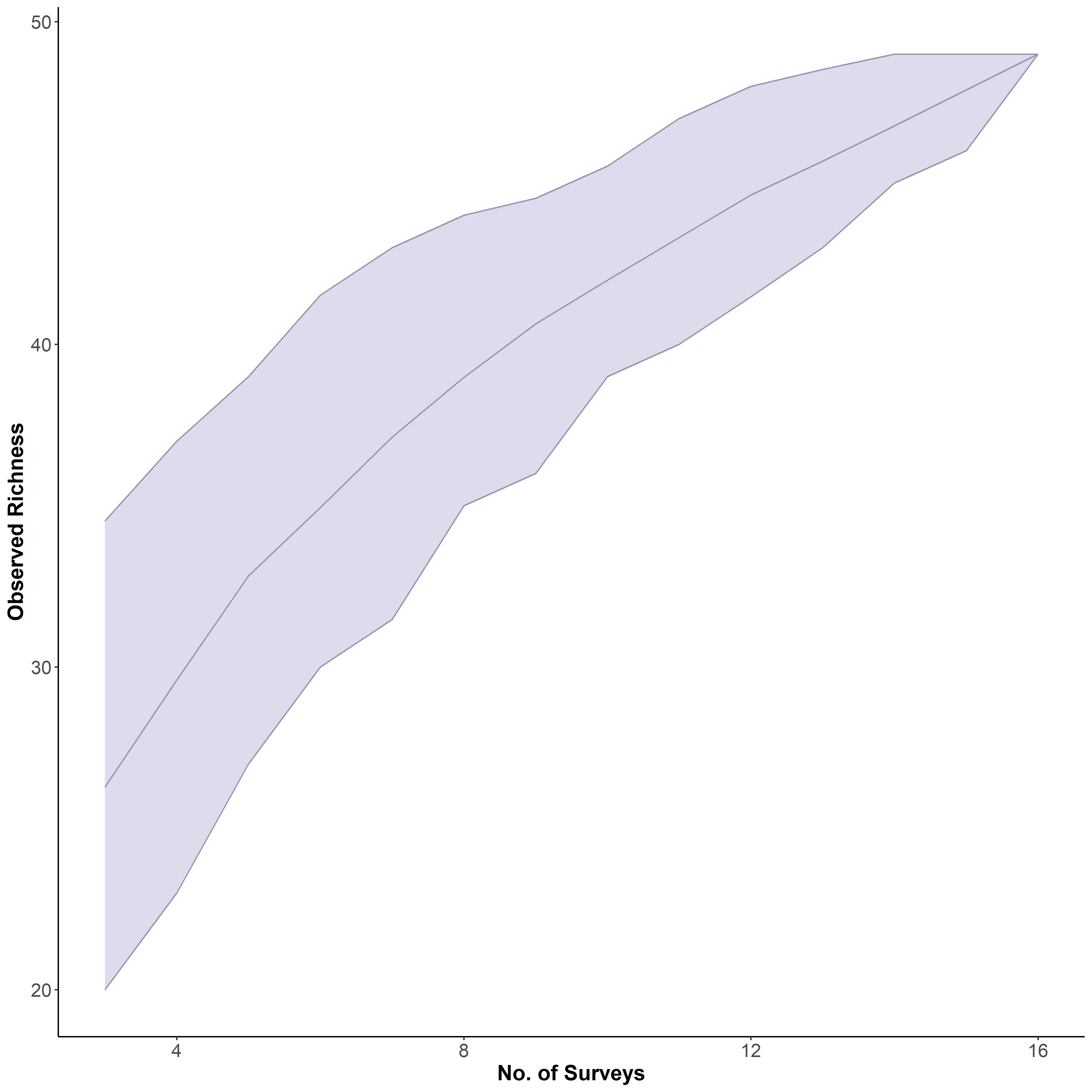

Supplement: Supplemental Information 3 — The curve did not reach a plateau after 16 surveys, showing the need for more replicates of the study. Light purple zone is the confidence interval around the mean with a level of confidence of 95%. [file peerj-12-17763-s003.png]
